# Papain-like protease of SARS-CoV-2 inhibits RLR signaling in a deubiquitination-dependent and deubiquitination-independent manner

**DOI:** 10.3389/fimmu.2022.947272

**Published:** 2022-08-12

**Authors:** Xiang-Hong Ran, Jia-Wu Zhu, Ya-Yun Chen, Run-Ze Ni, Dan Mu

**Affiliations:** ^1^ Institute of Life Sciences, Chongqing Medical University, Chongqing, China; ^2^ School of Basic Medical Sciences, Kunming Medical University, Kunming, China

**Keywords:** SARS-CoV-2, papain-like protease, RLR signaling, IFN, deubiquitination

## Abstract

The newly emerged severe acute respiratory syndrome (SARS) coronavirus-2 (SARS-CoV-2) can result in dysregulated interferon (IFN) responses that contribute to disease severity. The papain-like protease of SARS-CoV-2 (SCoV2-PLpro) has been previously reported to attenuate IFN responses, but the underlying mechanism is not fully understood. In this study, we found that SCoV2-PLpro potently suppressed IFN production and signaling induced by Sendai virus as well as RIG-I-like receptor (RLR) signaling pathway components, including RIG-I, MAVS, TBK1, TRAF3, TRAF6, and IRF3. SCoV2-PLpro exhibited different specificity and efficiency than SARS-CoV PLpro, with the former exerting a greater inhibitory effect on the RIG-I- and TRAF3-mediated IFN response but a weaker effect on the MAVS-mediated IFN response. Furthermore, we showed that SCoV2-PLpro significantly reduced K63-ubiquitination of RIG-I, MAVS, TBK1, TRAF3, TRAF6, and IRF3 and K48-ubiquitination of IκBα, which are known critical for the innate immune signal transduction. The deubiquitinating (DUB) activity of SCoV2-PLpro required a catalytic residue cysteine 111 (C111) but not the UBL domain. Notably, by utilizing the DUB-defective C111 mutant, we demonstrated that SCoV2-PLpro targeted RLR signaling pathway regulators *via* deubiquitination-dependent and -independent mechanisms, with the inhibitory activities of RIG-I and TBK1 correlating with DUB function, whereas the antagonism effects on MAVS, TRAF3, TRAF6, and IRF3 independent on DUB activity. Overall, our results reveal that SCoV2-PLpro evolves differential IFN antagonism activity from SCoV1-PLpro and it targets multiple key RLR signaling pathway components *via* various mechanisms, providing insights into SARS-CoV-2 pathogenesis and clues for developing antiviral therapies for COVID-19.

## Introduction

Severe acute respiratory syndrome coronavirus-2 (SARS-CoV-2) is a novel coronavirus that broke out in late 2019 and caused a highly contagious disease, coronavirus disease-2019 (COVID-19) that led to one of the most devastating global pandemics in history. SARS-CoV-2 belongs to the family Coronaviridae and genus Betacoronavirus, and is phylogenetically closely related to SARS-CoV and Middle East respiratory syndrome-coronavirus (MERS-CoV), two other highly pathogenic coronaviruses that emerged in 2002 and 2012, respectively (1). SARS-CoV-2 encodes 4 structural proteins (nucleocapsid [N], envelope [E], membrane [M], and spike [S] proteins), 16 nonstructural proteins (NSP1–NSP16, which includes an RNA polymerase, helicase, and other components required for viral replication), and 7 accessory proteins (ORF3a–ORF8). Among the genetic components, ORF1a encodes two cysteine proteases, a papain-like protease (PLpro) in the NSP3 family, which is critical for the proteolytic cleavage of NSP1-3, and a 3C-like protease (3CLpro; also known as the “main protease”) in NSP5, which activates the other 13 NSPs (2).

Eight human coronaviruses had been identified prior to the SARS-CoV-2-induced COVID-19 outbreak (2). These coronaviruses cause disease in humans ranging from the common cold (HCoV-229E, OC43, HCoV-NL63 and -HKU1) to SARS, which has led to 13% mortality among those infected, and MERS, which has caused 35% mortality in those infected, and are characterized by an aberrant immune responses that fail to elicit strong interferon (IFN) action or proinflammatory innate immunity (2–4). Generally, upon viral infection, the host immediately launches an innate immune response that is initiated by a number of cellular pattern recognition receptors (PRRs). Viral nucleic acids such as viral genes or replicative intermediates are recognized by two different classes of PRRs, i.e., membrane-bound Toll-like receptors (TLR3/7/8/9) and retinoid acid-inducible gene (RIG)-I-like receptors (RLRs), which include RIG-I and melanoma differentiation-associated gene 5 (MDA5). RIG-I/MDA5 binds to viral single-stranded RNA and/or double-stranded RNA (dsRNA) and transfer a signal to mitochondrial antiviral signaling protein (MAVS) (5). MAVS then recruits both tumor necrosis factor receptor-associated factor 3 (TRAF3) and TRAF6 to form the MAVS/TRAF3/TRAF6 signalosome (6). The signalosome transmits signals to downstream kinase complexes, such as IKKi and TBK1, which phosphorylate IRF3, inducing its dimerization and nuclear translocation. In the nucleus, IRF3 in conjunction with activated NF-κB induce the synthesis of IFN. The NF-κB pathway is also activated *via* these mediators. IKKα, IKKβ, and IKKγ kinases phosphorylate the NF-κB inhibitor IκBα, which is subsequently targeted for degradation, allowing NF-κB to be translocated into the nucleus. IFNs induce the activation of the STAT1 signaling pathway, which is characterized by the formation and nuclear translocation of the STAT1/STAT2/IRF9 complex. The complex binds to IFN-I-stimulated response elements (ISREs), inducing the expression of hundreds of IFN-stimulated genes (ISGs) that elicit antiviral effects. Innate immune signaling is regulated through ubiquitination at multiple levels. For example, RIG-I, MAVS, TBK1/IKKi, TRAF3 and TRAF6 are activated through K63-linked ubiquitination, while IKKα/IKKβ-induced degradation of IκBα relies on K48-linked ubiquitination. These ubiquitin-mediated regulations pose an excellent opportunity for viral immune evasion through the deconjugation of ubiquitin mediated by deubiquitinating (DUB) enzymes (7).

Coronaviruses have been reported to induce a range of strategies to suppress host innate immune responses, including antagonizing IFN production, inhibiting IFN signaling, and enhancing IFN resistance (8). The cysteine PLpro, which is known to hydrolyze peptide and isopeptide bonds in viral and cellular substrates, has been found to possess DUB, de-ISGylating and IFN antagonizing activities. PLpro in SARS-CoV, MERS-CoV, HCoV-NL63, and murine CoV has been implicated in deubiquitination-based innate immune evasion (9–13). SARS-CoV-PLpro (hereafter, SCoV1-PLpro) has been reported to remove K63-linked polyubiquitinated chains from RIG-I, STING, TBK1, IRF3, TRAF3, and TRAF6 (11, 13, 14) and to stabilize IκBα by inhibiting K48 linked-ubiquitination-mediated degradation (15). DUB activity has been reported to depend on an intact catalytic cysteine residue located at amino acid (aa) position 1651 in SARS-CoV, aa 1678 in NL63-CoV, and aa 1592 in MERS-CoV (9, 10, 16–18). A previous study by Shin et al. demonstrated that SCoV2-PLpro preferentially cleaved the ubiquitin-like protein ISG15 and attenuated type I IFN responses after stimulation by IFNα and TNFα, thus impeding the nuclear translocation of IRF3 and NF-κB, and that a catalytically inactive mutation at aa C111 in SCoV2-PLpro (corresponding to aa C1651 in SARS-CoV) diminished de-ISGylating activity (19). Liu et al. also reported that SCoV2-PLpro inhibited ISG15-mediated MDA5 signaling *via* its de-ISGylase activity (20). The DUB activity of SCoV2-PLpro has been demonstrated in several studies (19, 21, 22), but whether or how SCoV2-PLpro regulates RLR signaling pathway through its DUB function remains unclear.

In the present study, we analyzed the DUB activity and IFN antagonism profile of the 315-aa core catalytic domain of papain-like protease in the newly emerged SARS-CoV-2 virus and compared it to that of SCoV1-PLpro. We found that SCoV2-PLpro potently suppressed IFN responses mediated by multiple key RLR signaling pathway components. We also showed that the effects of SCoV2-PLpro differed from those of SCoV1-PLpro in a signaling-molecule-dependent manner. Furthermore, we showed that SCoV2-PLpro significantly impaired K63-ubiquitination of RIG-I, MAVS, TBK1, TRAF3, TRAF6, and IRF3 and K48-ubiquitination of IκBα. By utilizing a C111 catalytic mutant which led to a loss of DUB effect, we demonstrated that SCoV2-PLpro targeted RLR signaling pathway regulators in a deubiquitination-dependent and -independent manner. This study highlights how PLpros from two closely related SARS coronaviruses can possess different specificities and efficiencies and extends the understanding of how SARS-CoV-2 utilizes one protease for targeting multiple RLR signaling pathway components *via* various mechanisms.

## Materials and methods

### Cell culture and antibodies

Human embryonic kidney 293T cells were maintained in Dulbecco’s modified Eagle’s medium supplemented with 10% fetal bovine serum (FBS) and tested for mycoplasma contamination periodically by a PCR-based mycoplasma detection kit (Beyotime Biotec).

For immunoblot analysis, the following antibodies were used: Mouse anti-Flag (sc-7392, Santa Cruz Biotechnology), Rabbit anti-HA (H6908, Sigma-Aldrich). Rabbit anti-Myc (16286-1), anti-V5 (14440-1), Mouse anti-GFP (66002-1), Mouse anti-GAPDH (60004-1), Mouse anti-Tubulin (66031-1), goat anti-mouse IgG-HRP and goat anti-rabbit IgG-HRP were from Proteintech. Rabbit anti-Flag (14793S), anti-HA (3724S), anti-p65 (8284S), anti-IRF3 (11904T) were purchased from Cell Signaling Technology. Rabbit anti-STAT1 (AF0288) and anti-LaminB1 (AF5222) were from Beyotime Biotec. Mouse anti-HA (201113) and anti-β-Actin (200068-8F10) antibodies were purchased from Zen-Bioscience.

### Plasmid construction

To obtain high expression in eukaryote cells, the codon usage of SCoV1-PLpro core domain (amino acids 1018 to 1277) and SCoV2-PLpro core domain (amino acids 1570 to 1884) were optimized based on human codon usage frequency, tagged with a Flag peptide, and cloned into pCDH-EF1-MCS-T2A-puro vector by BamHI and NotI. pCDH-SCoV2-PLpro-C111A-Flag was generated by the point-mutation method. Primers as follows: BamHI-PLpro-For: CGGGATCCGCCACCATGGAGG, PLpro-NotI-Rev: TTTTCCTTTTGCGGCCGCTCACTTGTCG, SCoV2-PLpro-C111A-For: GTGGGCCGACAACAACCGATACCTGGCCACCGCCC, SCoV2-PLpro-C111A-Rev: GGGCGGTGGCCAGGTATCGGTTGTTGTCGGCCCAC. The pCDH-SCoV2-PLpro-ΔUBL-Flag were constructed by PCR amplification of the corresponding DNA fragment from SCoV2-PLpro-Flag with the primers BamHI-ΔUBL-PLpro2-For: CGGGATCCGCCACCATGCCCAATGACGACACCCTG and PLpro-NotI-Rev. The pLPCX vectors encoding hemagglutinin (HA)-tagged N-terminal RIG-I, MAVS, TBK1, TRAF6, and V5-tagged TRAF3 were generated by PCR amplification in human cDNA with HA or V5 tagged primers. The following primers were used: N-RIG-I (Forward primer: CCGCTCGAGgccaccATGACCACCGAGCAGCGACG, Reverse primer: TAAGATGATGTTCACATATAAGCAG), TBK1 (Forward primer: CCGCTCGAGGCCACCATGCAGAGCACTTCTAATCATC, Reverse primer: CTAAAGACAGTCAACGTTGCG), TRAF3 (Forward primer: CCGCTCGAGGCCACCATGGAGTCGAGTAAAAAGATGG, Reverse primer: TCAGGGATCGGGCAGATCCGAAG), TRAF6 (Forward primer: CCGCTCGAGGCCACCATGAGTCTGCTAAACTGTG, Reverse primer: CTATACCCCTGCATCAGTAC), MAVS (Forward primer: CCGCTCGAGGCCACCATGCCGTTTGCTGAAGACAAGACCTATAAG, Reverse primer: CTAGTGCAGACGCCGCCGGTACAG). pEGFP-C3-IRF3 was purchased from PPL (Public Protein/Plasmid Library, China). pEGFP-C3-IRF3-5D was generated by overlapping PCR and cloned into pEGFP-C3 by Xhol and Kpnl. The primers used were: XhoI-IRF3-5D-For: CCGCTCGAGGCCACCATGGGAACCCCAAAGC, pEGFP-C3-IRF3-KpnI-Rev: GGCGCGGTACCGTCGACT, IRF3-5D-Mut-For: GTGGACCTGCACATTGATAACGATCACCCACTCGATCTCGATGACGACCAGTACAAGGC, IRF3-5D-Mut-Rev: GCCTTGTACTGGTCGTCATCGAGATCGAGTGGGTGATCGTTATCAATGTGCAGGTCCAC. pGL4.32-NF-κB-luc, pGL4.32-IFN-β-luc, pGL4.32-ISRE-luc, pRL-TK (*Renilla* luciferase) and Myc-tagged wild type ubiquitin (WT-Ub-Myc) were kindly provided by Dr. Yong-Tang Zheng (Kunming Institute of Zoology, Chinese Academy of Sciences), K48-Ub-Myc and K63-Ub-Myc were synthesized and replaced WT-Ub in the original plasmid by BglII and XhoI.

### Co-immunoprecipitation and western blot

To extract the whole-cell lysates, 293T cells were co-transfected with Flag-tagged PLpro plasmid together with Myc-tagged ubiquitin expressing plasmid using Lipofectamine 2000, cells were treated with SeV (MOI=1) for 8 hrs. After 48 hrs post-transfection, cells were harvested and washed twice with PBS, then lysed with RIPA lysis buffer (20mM Tris [pH7.5], 150 mM NaCl, 1% Triton X-100) with 1 mM phenylmethyl sulfonyl fluoride (Beyotime Biotec) and protease inhibitor cocktail (Thermo Fisher Scientific, USA). For nuclear and cytoplasmic fractionation, 293T cells transfected with the indicated plasmids for 36 hrs and then treated with SeV for 8 hrs were processed using NE-PERTM Nuclear and Cytoplasmic Extraction Reagents (catalog no.78833; Thermo Fisher Scientific, USA) according to the manufacturer’s protocol.

Cell lysates were separated by sodium dodecyl sulfate-polyacrylamide gel electrophoresis (SDS-PAGE) and then transferred to polyvinylidene fluoride membranes (Millipore). The blots were probed with indicated primary antibody, followed by an IgG-peroxidase-conjugated secondary antibody. Afterwards, the blots were illuminated with chemiluminescent detection reagents (Millipore).

For all the ubiquitination-related co-immunoprecipitation (Co-IP), different tagged protein expression constructs were co-transfected with K48-Ub-myc or K63-Ub-myc plasmids into 293T cells together with or without WT-PLpro or PLpro mutant plasmid in 6-cm dish. After treatment with SeV (MOI=1) for 8 hrs, cells were washed twice with PBS and lysed in 500 μl RIPA lysis buffer with 1 mM phenylmethyl sulfonyl fluoride and protease inhibitor cocktail. Cell lysates were centrifuged at 12,000 × g for 10 min, and 450 μl of the clarified supernatant was used for immunoprecipitation; the remaining 50 μl lysate was diluted in 5× SDS-PAGE sample buffer, incubated at 100°C for 5 min. Rabbit anti-HA, rabbit anti-V5, or mouse anti-GFP was conjugated to 20 μl of Protein A/G-agarose beads (Santa Cruz Biotechnology) and incubated for 1 h at room temperature. The beads were centrifuged for 1 min at 1,000 × g, washed three times with 1 ml lysis buffer by spinning, added to 450 μl cell extract, and then incubated overnight at 4°C. Beads were washed four times with 1 ml lysis buffer and the pellet was resuspended in 25 μl 2× SDS-PAGE sample buffer.

### Dual-luciferase assay

Transfection of 293T cells with SCoV1-PLpro, SCoV2-PLpro, SCoV2-PLpro mutant plasmids (100 ng) or empty vector pCDH (100 ng) and indicated different signaling molecule plasmids (100 ng), together with plasmid DNA expressing firefly luciferase that is under the control of various transcriptional response element, including NF-κB-luc (100 ng), IFN-β-luc (100 ng) and ISRE-luc (100 ng), was conducted using Lipofectamine 2000 reagents (Invitrogen) as described by the manufacturer’s instructions. In addition, a pRL-TK reporter plasmid was added to each transfection for normalization of transfection efficiencies. Cells were treated with or without SeV for 8 hrs and harvested at 36 hrs post-transfection. Luciferase activities were determined using a Dual-Luciferase Reporter Assay System (Promega) according to the protocol.

### RNA isolation, reverse transcription and qPCR

293T cells were transfected with SCoV1-PLpro, SCoV2-PLpro or SCoV2-PLpro mutant plasmids. Cells were treated by SeV for 8 hrs at 40 hrs post-transfection. Total cellular RNA was then extracted using RNAiso Plus reagent (TaKaRa) and reverse transcribed into cDNA using the RevertAid First Strand cDNA Synthesis Kit (Thermo Fisher Scientific, USA). Real-time PCR was performed on a Bio-Rad CFX96 real-time PCR system. The primers used were: *CXCL10* (Forward primer: TCCACGTGTTGAGATCATTGC, Reverse primer: TCTTGATGGCCTTCGATTCTG), *IL-6* (Forward primer: GTGAGGAACAAGCCAGAG, Reverse primer: GACCAGAAGAAGGAATGC), *IFNB1* (Forward primer: CCAACAAGTGTCTCCTCCAAAT, Reverse primer: AATCTCCTCAGGGATGTCAAAG), *ISG56* (Forward primer: CCTGAAAGGCCAGAATGAGG, Reverse primer: TCCACCTTGTCCAGGTAAGT), *GAPDH* (Forward primer: GCTTCGCTCTCTGCTCCTCCTGTT, Reverse primer: ACGACCAAATCCGTTGACTCCGACC). Amplifications were run as follows: initial activation was at 95°C for 2 min, and the subsequent 40 cycles in two phases consisted of 95°C for 15 s and 60°C for 30 s. The expression level was normalized with GAPDH.

### Statistical analysis

Data were analyzed as means ± SD. The statistical significance analyses were performed using two-sided unpaired t test (P values). Analyses were performed by using Prism 8 software (GraphPad). A significant difference was scored as * p< 0.05, ** p<0.01, *** p <0.001 and **** p <0.0001.

## Results

### SCoV2-PLpro impairs the activation of NF-κB-, IFN-β- and ISRE-responsive signaling pathways

**Figure 1:**
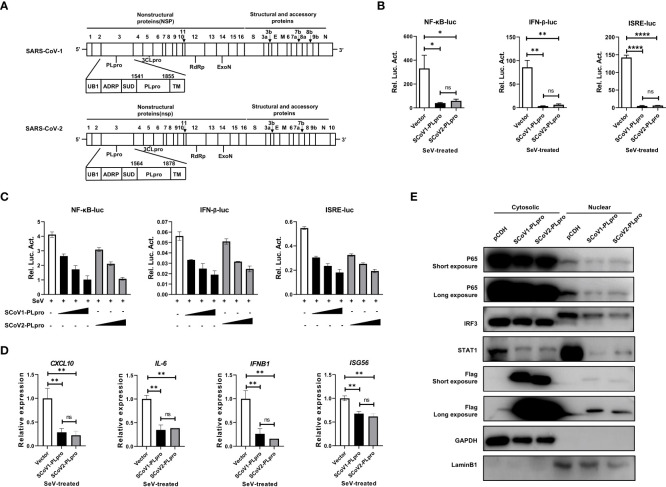
SARS-CoV-1 and SARS-CoV-2 papain-like proteases impair the activation of NF-κB-, IFN-β- and ISRE-responsive signaling pathways. **(A)** Schematic diagram showing the SARS-CoV-1 and SARS-CoV-2 genomic structures. The expanded regions below show the nonstructural protein PLpro. **(B)** Plasmids encoding SCoV1-PLpro, SCoV2-PLpro or an empty vector as the negative control were cotransfected into 293T cells with NF-κB-luc, IFN-β-luc or ISRE-luc constructs. A pRL-TK plasmid was transfected to normalize the transfection efficiency data. Luciferase activity was measured in cell lysates treated with Sendai virus (SeV) (multiplicity of infection [MOI] = 1) for 8 hrs starting 36 hrs post-transfection. **(C)** Increasing amounts of plasmid encoding SCoV1-PLpro or SCoV2-PLpro were cotransfected into 293T cells with NF-κB-luc, IFN-β-luc or ISRE-luc constructs. Luciferase activity was measured in cell lysates treated with SeV (MOI = 1) for 8 hrs starting 36 hrs post-transfection. **(D)** qPCR analysis of *CXCL10*, *IL-6*, *IFNB1* and *ISG56* mRNA expression in 293T cells transfected with SCoV-PLpro-carrying vectors for 36 hrs and then treated with SeV (MOI = 1) for 8 hrs. **(E)** The nuclear transport of activated p65, IRF3 and STAT1 was compared in SCoV1-PLpro-, SCoV2-PLpro- and pCDH empty vector-transfected 293T cells treated with SeV (MOI = 1) for 8 hrs. Immunoblotting was performed with rabbit anti-p65, anti-IRF3, anti-STAT1 and anti-Flag antibodies. The data are representative of three independent experiments and are reported as the mean ± SD (n = 3). Statistical significance analyses were performed with two-sided unpaired t tests **(B**, **D)**. *P < 0.05; **P < 0.01; ****P < 0.0001; and ns, not significant.

PLpro domains in SARS-CoV-1 and SARS-CoV-2 are encoded by NSP3, and they have a similar constitution and overall structure (**Figure 1A**) (21, 23). To evaluate and compare the activities of these two closely related PLpros in regulating the RNA-triggered innate antiviral immune response, we first employed NF-κB-, IFN-β- and ISRE-responsive luciferase (luc) reporter systems in the context of Sendai virus (SeV, an RNA virus that strongly activates RLR signaling) stimulation. Each reporter plasmid was cotransfected with a synthetic, codon-optimized parent construct encoding SCoV1-PLpro (aa residues 1541 to 1855) or SCoV2-PLpro (aa residues 1564 to 1878) in frame with a Flag epitope tag with *Renilla* luciferase into 293T cells. Consistent with previous reports, SCoV1-PLpro exerted a profound suppressive effect on the innate immune response, significantly inhibiting the SeV-induced activation of the NF-κB-luc, IFN-β-luc and ISRE-luc reporter genes (**Figure 1B**). Notably, SCoV2-PLpro showed suppressive effects comparable to that of SCoV1-PLpro on the induction of NF-κB-luc, IFN-β-luc and ISRE-luc activation (**Figure 1B**). Furthermore, SCoV2-PLpro downregulated SeV-induced activation of all tested reporter genes in a dose-dependent manner (**Figure 1C**).

Next, we assessed the effect of PLpros on the mRNA expression of the *CXCL10*, *IL6*, *IFNB1*, and *ISG56* genes induced by SeV infection. A qPCR analysis indicated that ectopic expression of both SCoV1-PLpro and SCoV2-PLpro inhibited the mRNA expression levels of the indicated genes upon SeV treatment. Consistent with the reporter assay results, SCoV2-PLpro exhibited an effect similar to that of SCoV1-PLpro in inhibiting *CXCL10*, *IL6*, *IFNB1* and *ISG56* expression (**Figure 1D**). For confirmation, we examined whether SCoV2-PLpro interferes with the nuclear translocation of IRF3, p65 and STAT1 as potent as SCoV1-PLpro. The findings showed that the nuclear import of all these transcription factors stimulated by SeV infection was greatly decreased in SCoV1-PLpro-transfected 293T cells compared with that in the vector control group. In addition, the potency of the SCoV2-PLpro suppressive effect on the SeV-stimulated nuclear translocation of IRF3, p65, and STAT1 was similar to that of SCoV1-PLpro (**Figure 1E**). Notably, a small portion of both SCoV1-PLpro and SCoV2-PLpro was translocated into the nucleus (**Figure 1E**), indicating a potential role for these proteins in the cell nucleus. Collectively, these data suggest that SCoV2-PLpro affects SeV-induced IFN production and downstream IFN signaling potently similar to that of SCoV1-PLpro.

### SCoV2-PLpro inhibits RLR signaling mediated by multiple key regulators

**Figure 2:**
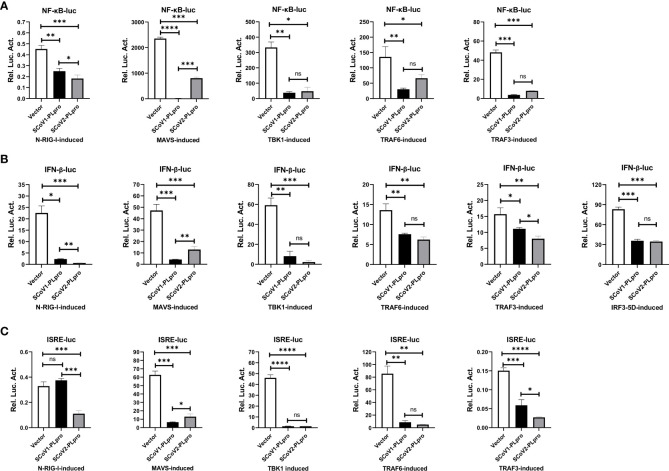
Suppression of N-RIG-I-/MAVS-/TBK1-/TRAF6-/TRAF3-/IRF3-5D-induced NF-κB-/IFN-β-/ISRE-luciferase promoter activation by SCoV1-PLpro and SCoV2-PLpro was assessed and compared. **(A–C)** 293T cells were cotransfected with the SCoV1-PLpro, the SCoV2-PLpro or a pCDH vector with the indicated plasmid and the NF-κB-luc **(A)**, IFN-β-luc **(B)** or ISRE-luc **(C)** construct. Twenty-four hours post-transfection, the cells were harvested, and a dual luciferase assay was performed. The data are representative of three independent experiments and are reported as the mean ± SD (n = 3). Statistical significance analyses were performed with two-sided unpaired t tests. *P < 0.05; **P < 0.01; ***P < 0.001; ****P < 0.0001; and ns, not significant.

We next investigated the step at which PLpro inhibits RLR signaling pathway and whether the two closely related SARS-CoV PLpros induce different effects. We asked whether key components, including RIG-I, MAVS, TBK1, TRAF6, TRAF3, and IRF3, which are crucial to RLR signaling, are targets of SCoV2-PLpro inhibition. Reporter assays showed that both SARS-CoV PLpros inhibited the activation of the NF-κB-luc, IFN-β-luc and ISRE-luc reporters mediated by overexpression of N-RIG-I (a constitutively active N-terminal portion of RIG-I), MAVS, TBK1, TRAF6, TRAF3, and IRF3-5D (a phosphorylated mimic of the activated form of IRF3) in 293T cells (**Figure 2A-C**). Notably, although both PLpros impeded TBK1-, TRAF6-, and IRF3-5D-induced IFN responses and signaling to a similar degree, the suppressive effect induced by SCoV2-PLpro on N-RIG-I-induced NF-κB-luc, IFN-β-luc and ISRE-luc activation was greater than that by SCoV1-PLpro, but its effect on downregulating MAVS-mediated activation of the same reporter genes was weaker. In addition, TRAF3-induced IFN-β and IRSE reporter activity was reduced to a greater extent in the presence of SCoV2-PLpro. Overall, these results suggest that PLpros from two closely related SARS coronaviruses possess different specificities and efficiencies in the suppression of innate immune defense responses mediated by various signaling molecules.

### SCoV2-PLpro antagonizes K48-linked ubiquitination of IκBα and K63-linked ubiquitination of RIG-I, MAVS, TBK1, TRAF6, TRAF3 and IRF3

**Figure 3:**
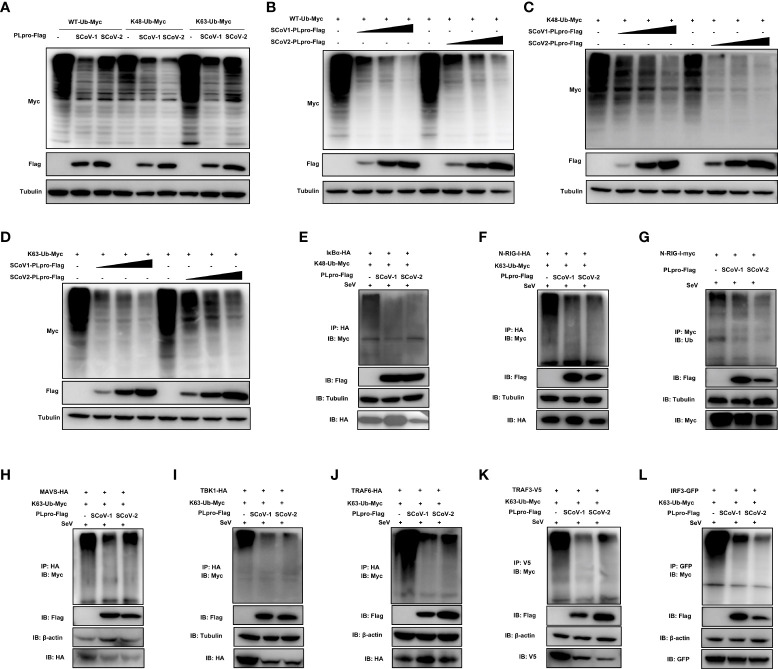
Characterization of the deubiquitinating (DUB) activity of SCoV2-PLpro. **(A)** SCoV1-PLpro-Flag, SCoV2-PLpro-Flag or a pCDH vector was cotransfected with WT-ubiquitin (Ub)-Myc, K48-Ub-Myc or K63-Ub-Myc into 293T cells. The level of ubiquitination was measured by western blot with anti-Myc antibody and anti-Flag antibodies. **(B–D)** Increasing amounts of plasmid encoding SCoV1-PLpro-Flag or SCoV2-PLpro-Flag were cotransfected with WT-ubiquitin (Ub)-Myc, K48-Ub-Myc or K63-Ub-Myc into 293T cells. The level of ubiquitination was measured by western blot with anti-Myc antibody and anti-Flag antibodies. **(E-L)** 293T cells were cotransfected with Flag-tagged SCoV-PLpro with Myc-tagged K48-Ub or K63-Ub and hemagglutinin (HA)-tagged IkBα **(E)**, N-RIG-I **(F)**, MAVS **(H)**, TBK1 **(I)**, TRAF6 **(J)**, Myc-tagged N-RIG-I **(G)**, V5-tagged TRAF3 **(K)** or GFP-tagged IRF3 **(L)** in 6-cm dishes. The cells were treated with SeV (MOI = 1) for 8 hrs starting 36 hrs post-transfection and then harvested and subjected to coimmunoprecipitation with an anti-HA antibody **(E, F, H, I, J)**, anti-Myc antibody **(G)**, anti-V5 antibody **(K)** or anti-GFP antibody **(L)**, followed by western blot with an anti-Myc or anti-Ub antibody to detect ubiquitination.

Successful innate immune signaling requires proper ubiquitination that leads to the activation of the key signaling molecules. We first characterized the DUB property of SCoV2-PLpro by examining its DUB effect on wild-type (WT) and specific K48- and K63-linked ubiquitinated proteins. 293T cells were cotransfected with plasmids encoding each PLpro protein with Myc-tagged ubiquitin. The decrease in protein ubiquitination conferred by PLpros was analyzed by western blot. As shown in **Figures 3A**-**D**, SCoV2-PLpro exhibited potent deubiquitination of the global protein and specific DUB effects on K48- and K63-linked ubiquitinated proteins in a dose-dependent manner, and its DUB efficiency against WT and K63-linked ubiquitinated chains was weaker than that of SCoV1-PLpro. In contrast, SCoV2-PLpro outperformed SCoV1-PLpro in removing K48-linked ubiquitin chains (**Figures 3A**, **C**).

Next, we investigated how SCoV2-PLpro and SCoV1-PLpro affected the K48-/K63-linked ubiquitination of the key players of innate immune responses. The results showed that the K48 ubiquitination of IκBα was greatly and equally eliminated by the presence of SCoV2-PLpro or SCoV1-PLpro (**Figure 3E**). In addition, SCoV2-PLpro exhibit profound DUB activity of removing the K63-linked polyubiquitin chains from all signaling molecules tested including N-RIG-I, MAVS, TBK1, TRAF6, TRAF3 and IRF3 (**Figures 3F**–**L**). These two SARS PLpros decreased K63- and the overall ubiquitination of N-RIG-I and K63-linked ubiquitinated chains of TRAF3 at a similar level (**Figures 3F**, **G**, **K**). Because SCoV2-PLpro was more efficient than SCoV1-PLpro in inhibiting IFN-β-luc and ISRE-luc signaling activated by N-RIG-I and TRAF3 (**Figures 2B, C**), a mechanism in addition to DUB-based evasion may underlie SCoV2-PLpro-mediated N-RIG-I and TRAF3 activity suppression. Moreover, consistent with the luciferase-based reporter assay (**Figures 2A–C**), SCoV2-PLpro downregulated K63 ubiquitination of MAVS less efficiently than SCoV1-PLpro and showed comparable DUB activity on K63-linked ubiquitinated chains of TBK1, TRAF6 and IRF3 (**Figures 3H–J, L**). Collectively, these data indicate that both SCoV2-PLpro and SCoV1-PLpro exhibit potent DUB activity against RLR signaling axis, leading to diverse effects in a signaling-molecule-specific manner.

### Mutation of a catalytic cysteine residue or deletion of the UBL domain does not abolish SCoV2-PLpro IFN antagonism

**Figure 4:**
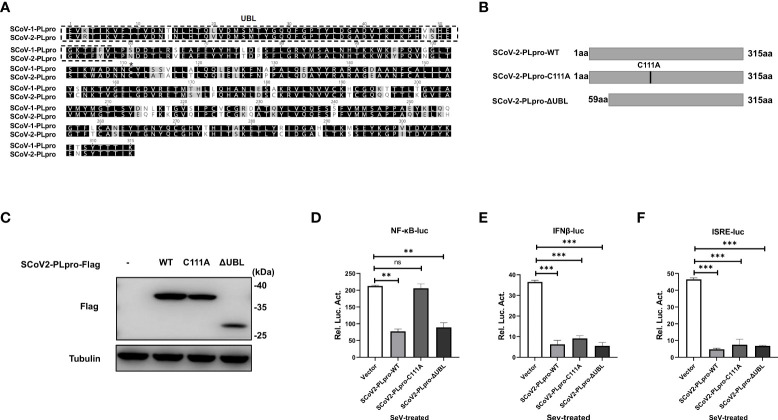
Effects of the SCoV2-PLpro-C111A and ΔUBL mutants on RLR signaling pathway. **(A)** Alignment of amino acid sequences of SCoV1-PLpro and SCoV2-PLpro. The UBL domain is highlighted by a dashed box. Asterisk indicates the conserved cysteine at residue 111. **(B)** Schematic diagram showing the point mutation (C111A) and domain-deletion mutation (ΔUBL) in SCoV2-PLpro. **(C)** Ectopic expression levels of SCoV2-PLpro-WT, C111A and ΔUBL were determined by western blot. **(D–F)** 293T cells were cotransfected with SCoV2-PLpro-WT, SCoV2-PLpro-C111A or SCoV2-PLpro-ΔUBL with NF-κB-luc **(D)**, IFN-β-luc **(E)** or ISRE-luc **(F)**. A pRL-TK plasmid was transfected to normalize the transfection efficiency data. Thirty-six hours post-transfection, the cells were treated with SeV (MOI = 1) for 8 hrs, and then luciferase activity was measured. The data are representative of three independent experiments and are the mean ± SD (n = 3). Statistical significance analyses were performed with two-sided unpaired t tests. **P < 0.01; ***P < 0.001; and ns, not significant.

The PLpro domain in SARS-CoV comprises an intact catalytic triad, a zinc-binding domain, and an N-terminal UBL domain. The Cys residue in the catalytic triad “Cys-His-Asp” is essential for the DUB activity of the SCoV1-PLpro domain (16); however, reports on the role played by the UBL domain in the SCoV1-PLpro antagonism of IFN responses are controversial (10, 15). SCoV2-PLpro encodes a conserved Cys at residue 111 and a UBL domain similar to that in SCoV1-PLpro (**Figure 4A**). To determine whether SCoV2-PLpro IFN antagonism depends on a catalytic site or the UBL domain, we constructed a C111A mutant, which had been previously shown to be catalytically inactive in SARS-CoV-2 (19), and a UBL-deletion mutant (**Figure 4B**). The ectopic expression of SCoV2-PLpro and mutants were verified by western blot (**Figure 4C**). 293T cells were transfected with the wild type of SCoV2-PLpro or one of the mutants, *Renilla* luciferase, and either NF-κB-luc, IFN-β-luc or ISRE-luc reporter plasmids. SeV was used to stimulate the expression of the corresponding reporters 24 hrs post-transfection. The results showed that the SCoV2-PLpro catalytic mutant had completely lost the ability to inhibit NF-κB reporter activity and showed an intact IFN antagonism activity (**Figures 4D–F**). In contrast, deletion of the UBL domain reversed the full antagonism against SeV-mediated NF-κB activation and IFN induction (**Figures 4D**–**F**). Collectively, these data indicate that the UBL domain does not contribute to SCoV2-PLpro IFN antagonism and that the catalytic C111 is responsible for SCoV2-PLpro NF-κB antagonism.

### Residue C111, but not the UBL domain, is indispensable for the SCoV2-PLpro-mediated DUB effect on key signaling regulators

**Figure 5:**
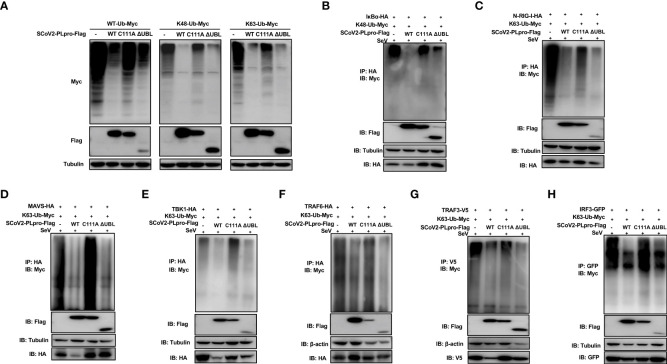
Effects of wild-type (WT) SCoV2-PLpro and mutants on ubiquitinated key regulators in RLR signaling pathway. **(A)** 293T cells were cotransfected with Flag-tagged WT or mutant SCoV2-PLpro plasmids with WT-Ub-Myc, K48-Ub-Myc or K63-Ub-Myc. The level of ubiquitination was measured by western blot with anti-Myc and anti-Flag antibodies. **(B–H)** WT or mutant SCoV2-PLpro-Flag plasmids were cotransfected with Myc-tagged K48-Ub or K63-Ub and HA-tagged IκBα **(B)**, N-RIG-I **(C)**, MAVS **(D)**, TBK1 **(E)**, TRAF6 **(F)**, V5-tagged TRAF3 **(G)** or GFP-tagged IRF3 **(H)** into 293T cells in 6-cm dishes. Thirty-six hours post-transfection, the cells were treated with SeV (MOI = 1) for 8 hrs and harvested and subjected to immunoprecipitation with anti-HA antibody **(B–F)**, anti-V5 antibody **(G)** or anti-GFP antibody **(H)**, followed by western blot using anti-Myc antibody and anti-Flag antibody to detect ubiquitination.

We next examined whether the C111A mutant or UBL deletion mutant interferes with the ubiquitination of key signaling regulators. First, plasmids encoding WT PLpro or each mutant were cotransfected with the WT-, K48-, or K63-linked ubiquitin plasmids. We observed that the introduction of the C111A mutation resulted in severely reduced ability of removing all types of ubiquitin chains tested, while the UBL-deletion mutant exhibited a DUB effect comparable to WT SCoV2-PLpro (**Figure 5A**). Next, SeV-induced K48-linked ubiquitination of IκBα and K63-linked ubiquitination of N-RIG-I, MAVS, TBK1, TRAF6, TRAF3, and IRF3 were assessed in the presence of WT or mutant SCoV2-PLpros. SCoV2-PLpro C111A showed severely impaired DUB activity against the K48-linked ubiquitinated chains on IκBα (**Figure 5B**), and it completely lost the ability to remove the K63-linked ubiquitin chains from MAVS, TBK1, TRAF6, and IRF3 (**Figures 5D–F**, **H**). However, a DUB effect on the K63-linked ubiquitination of N-RIG-I and TRAF3 retained to some extent (**Figures 5C**, **G**). In addition, the UBL-deletion SCoV2-PLpro mutant exhibited a different DUB activity pattern than the C111A mutant; specifically, the UBL-deletion mutant showed intact DUB activity against K63-linked ubiquitination of N-RIG-I, MAVS, TBK1, TRAF6, TRAF3, and IRF3 as well as intact DUB activity against K48-linked ubiquitination of IκBα (**Figure 5B**–**H**). Altogether, these results indicate that residue C111, but not the UBL sequence, is a main determinant for the SCoV2-PLpro-mediated DUB effect on the key signaling components.

### SCoV2-PLpro-mediated antagonism of MAVS-, TRAF6-, TRAF3-, and IRF3-induced IFN responses decouples DUB activity

**Figure 6:**
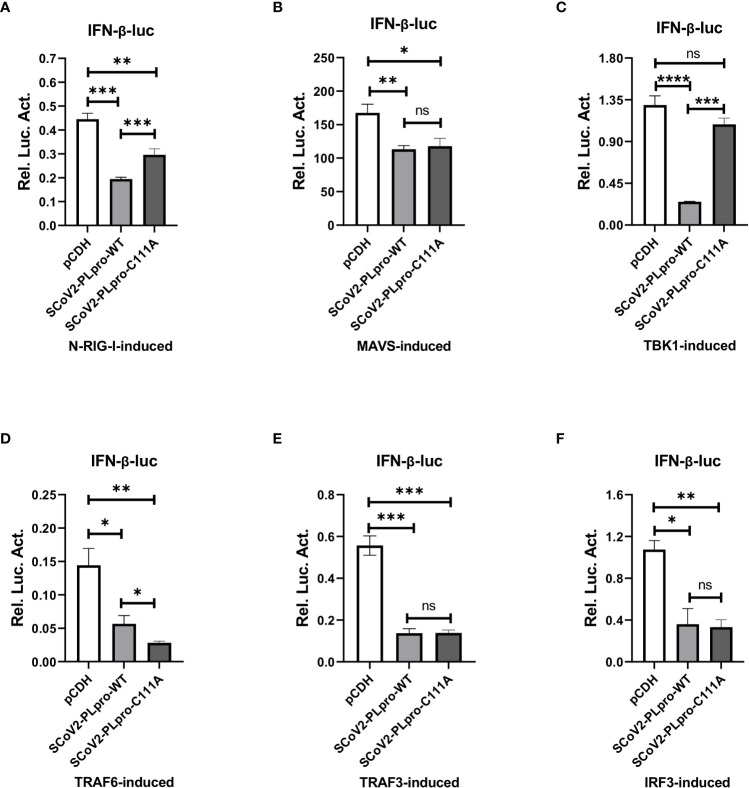
Residue C111, but not the UBL domain, is indispensable for the SCoV2-PLpro-mediated DUB effects on key signaling regulators. **(A–F)** 293T cells were cotransfected with IFN-β-luc and SCoV2-PLpro-Flag or SCoV2-PLpro C111A mutant. Plasmids expressing N-RIG-I-HA **(A)**, MAVS-HA **(B)**, TBK1-HA **(C)**, TRAF6-HA **(D)**, TRAF3-V5 **(E)** or IRF3-GFP **(F)** was transfected to activate the IFN expression pathway. A pRL-TK plasmid encoding *Renilla* luciferase was used to normalize the transfection efficiency data. The cells were transfected and incubated for 24 hrs. Luciferase activity was assayed with a dual-luciferase reporter assay. The data are representative of three independent experiments and are reported as the mean ± SD (n = 3). Statistical significance analyses were performed with two-sided unpaired t tests. *P < 0.05; **P < 0.01; ***P < 0.001; ****P < 0.0001; and ns, not significant.

**Figure 7:**
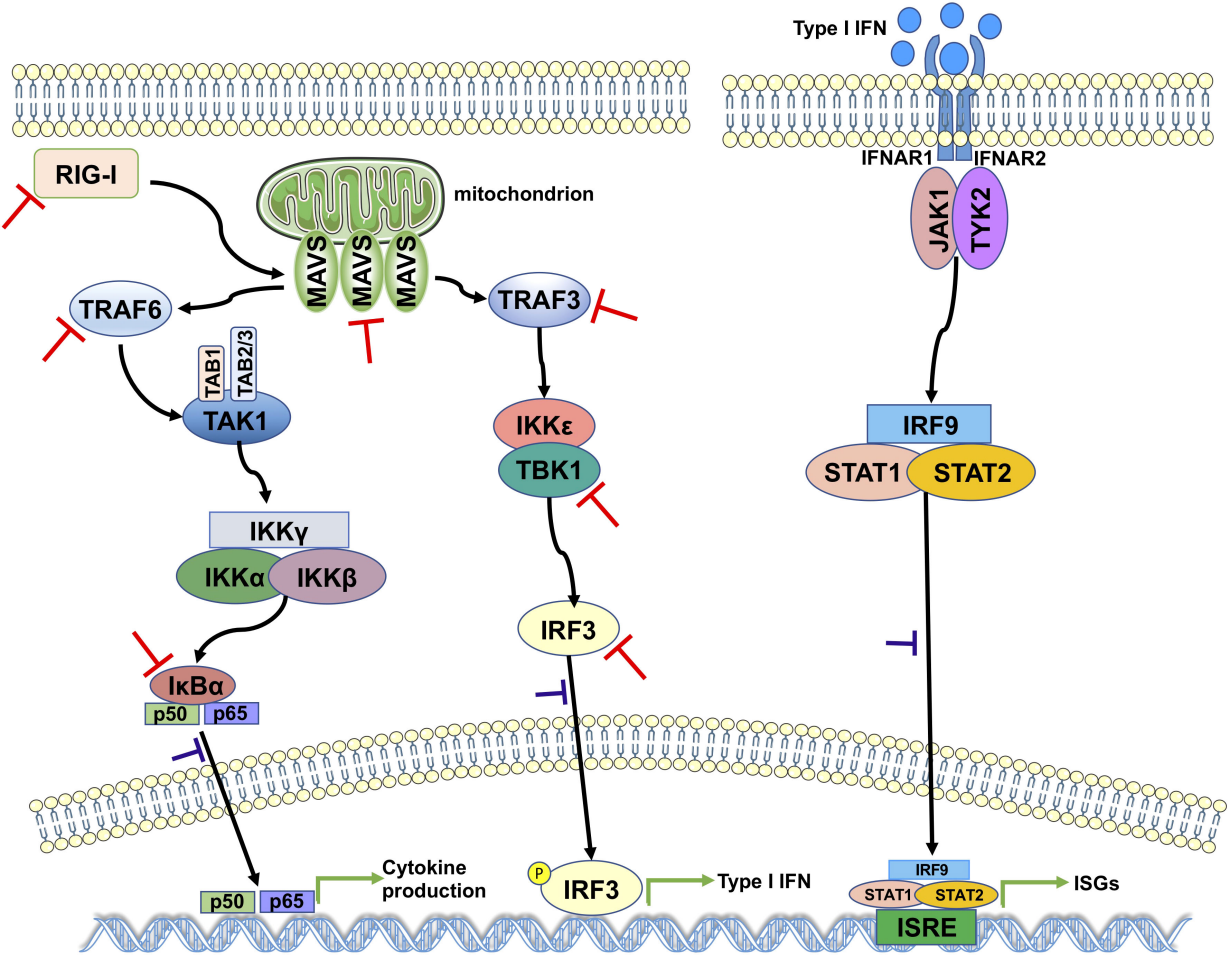
A proposed model for SCoV2-PLpro-mediated antagonism of innate immune signaling. SCoV2-PLpro inhibits IFN responses mediated by RIG-I, MAVS, TBK1, TRAF6, TRAF3, and IRF3 as well as the K48-ubiquitinated chain of IκBα (indicated by red inhibition lines), leading to impaired nuclear translocation of p65, IRF3, and STAT1 (indicated by purple inhibition lines).

The results of the aforementioned experiments showed that the catalytic C111 mutation resulted in substantially attenuated DUB activity but did not abrogate IFN antagonism. Therefore, we set out to elucidate the effect of DUB activity on the IFN responses induced by various key intermediates in RLR signaling pathway. A luciferase-based IFN-β promoter reporter assay was performed by cotransfecting the IFN-β-luc reporter, the WT SCoV2-PLpro or mutant plasmids, *Renilla* luciferase, and plasmids encoding either N-RIG-I, MAVS, TBK1, TRAF6, TRAF3, or IRF3-5D into 293T cells. The results revealed that the SCoV2-PLpro inhibition of the N-RIG-I-mediated IFN response was attenuated to some extent by the C111 mutation (**Figure 6A**), which correlated with the observation that C111A led to decreased DUB activity (**Figure 5C**). In addition, the C111A mutant, which completely abolished the DUB function against TBK1 K63-linked ubiquitinated chains, failed to inhibit TBK1-mediated IFN-β-luc activation (**Figure 6C**). However, the antagonism against the MAVS-, TRAF6-, TRAF3- and IRF3-5D-activated IFN-β promoter activity conferred by the C111A mutant was at a level similar to or even greater than that of the WT SCoV2-PLpro (**Figures 6B**, **D**–**F**), although this mutant exhibited full DUB activity against the ubiquitinated chains on these factors (**Figures 5D**, **F**–**H**). Taken together, our results revealed that SCoV2-PLpro targeted multiple key regulators in RLR signaling pathway *via* various mechanisms, such as by blocking RIG-I- and TBK1-mediated IFN induction in a DUB-dependent manner and MAVS-, TRAF3-, TRAF6- and IRF3-mediated IFN responses through DUB-independent mechanism(s) (**Figure 7**).

## Discussion

The COVID-19 pandemic has posed an unprecedented threat to global public health, making a thorough understanding of the molecular mechanisms underlying SARS-CoV-2 pathogenesis an urgent and necessary undertaking. SARS-CoV-2 can lead to a dysregulated innate immune response by manipulating signaling pathways (24). Previous studies have revealed that both SARS-CoV-2 structural and nonstructural proteins activate or counteract the antiviral action of IFN (25–30). To gain more insight into the interplay between SARS-CoV-2 and the host immune system, we characterized the IFN antagonism and DUB activity of the PLpro domain in SARS-CoV-2 on key components that are known to be activated by K48- or K63-linked polyubiquitination in RLR signaling pathway cascade, and we compared these characteristics with those we obtained for SCoV1-PLpro. We also determined that the DUB activity of SCoV2-PLpro contributes to the antagonism of IFN activation mediated by various signaling mediators.

The first report explaining the mechanism by which SCoV2-PLpro evades the innate immune response revealed that SCoV2-PLpro attenuated type I IFN responses activated by polyinosinic-polycytidylic acid (poly [I:C]), a synthetic double-stranded RNA that can mimic viral nucleic acids, and TNFα, a proinflammatory cytokine that activates NF-κB (19). Here, we demonstrated that SCoV2-PLpro dampened NF-κB activation and IFN induction stimulated by SeV, an RNA virus, as well as by a range of key components in RLR signaling pathway, including RIG-I, MAVS, TBK1, TRAF3, TRAF6 and IRF3. With a high identity (83%) at the aa level (22), SCoV1-PLpro and SCoV2-PLpro exhibited similar inhibitory features against SeV-induced NF-κB and IFN responses in this study. However, SCoV1-PLpro and SCoV2-PLpro exerted distinct antagonistic effect on IFN inductive activity mediated by various signaling molecules, such as RIG-I, MAVS, and TRAF3. These findings highlight that proteins in closely related viruses within the same species can function in notably divergent ways.

Ubiquitination plays an important role in the regulation of pathways involved in detecting and counteracting viral infections (7). A number of viruses have been found to possess DUB enzymes that manipulate these signaling processes by reversing the conjugation of ubiquitin on key regulators (31–33). Accumulating evidence has shown that PLpro domains in coronaviruses, arteriviruses, murine hepatitis virus, and porcine reproductive and respiratory syndrome virus are implicated in DUB-mediated innate immune evasion (13, 15, 17, 33–35). In this study, we showed that SCoV2-PLpro exerted a profound and global deconjugation effect on ubiquitinated host cell conjugates and that SCoV2-PLpro exhibited weaker DUB activity than SCoV1-PLpro against global ubiquitin-modified proteins and K63-linked ubiquitin chains, in agreement with a previous report (19).

The contribution of DUB activity to PLpro-mediated innate immune evasion remains controversial. Studies on PLpro in arteriviruses have shown that the abrogation of DUB activity significantly disrupted the ability of the virus to block IFN induction (31, 34). Bailey-Elkin and coworkers reported that MERS PLpro variants specifically lacking DUB activity did not exert IFN antagonistic effects (9). In contrast, the IFN antagonism by human coronavirus NL63 PLP2 did not require DUB function, as suggested by treatment with an inhibitor that blocks coronavirus DUB activity, which did not abrogate IFN antagonism (10). Mielech et al. demonstrated that SARS-CoV PLpro with a C1651A mutation failed to inhibit the mRNA expression of *CCL5*, *IFNB* and *CXCL10* (18); this mutant had been reported to be lacking DUB activity by Barretto and coworkers (16). Additionally, Matthews et al. showed that inhibition of SARS-CoV PLpro DUB function against on IRF3 ubiquitinated chains *via* mutagenesis abolished the IRF3 inhibitory activity of PLpro (14). In contrast, a DUB-independent mechanism that contributed to IFN antagonism has also been reported by Clementz and coworkers for SARS-CoV PLpro (10).

Here, we report that DUB activity largely depended on residue C111 in SCoV2-PLpro. However, although the loss of DUB activity through the introduction of the C111A mutation resulted in a complete abrogation of NF-κB suppression, the ability of the PLpro to block IFN activation was not abolished. This finding agrees with the study by Clementz et al. showing that SARS-CoV PLpro affected the expression of an NF-κB-dependent reporter but did not alter the antagonism against IRF3-dependent reporters (10). By investigating the effect of the C111 mutation on the ubiquitination of key regulators in RLR signaling pathway, we first found that residue C111 was critical for full SCoV2-PLpro DUB activity against the K48-linked ubiquitinated chains on IκBα and the K63-linked ubiquitinated chains on MAVS, TBK1, TRAF6 and IRF3. However, the C111 mutant retained certain DUB effect on removing the K63-linked ubiquitinated chains from RIG-I and TRAF3, indicating that there exists some other determinants in the SCoV2-PLpro sequence for deubiquitinating RIG-I and TRAF3. Correspondingly, the suppression of RIG-I- and TRAF3-activated IFN-β reporter expression was retained with the C111A mutant. Interestingly, even when the K63-linked ubiquitination of MAVS, TRAF6 and IRF3 was completely restored by the cystine mutation, SCoV2-PLpro C111A exerted antagonistic activity against MAVS-, TRAF6-, and IRF3-activated IFN-β reporter expression as potent as WT SCoV2-PLpro. These results strongly suggest an additional mechanism underlies SCoV2-PLpro-mediated MAVS, TRAF6, and IRF3 inhibition. We are currently working on delineating the DUB-independent mechanism by which coronavirus PLpros inhibit IFN induction.

The UBL domain did not contribute to the DUB or IFN antagonistic effect of SCoV2-PLpro in this study, which is consistent with the report by Clementz and coworkers indicating that the UBL domain in SARS-CoV PLpro exerted no effect during the antagonism of type I IFN induction (10), but is inconsistent with studies by Frieman et al. showing that deletion of the SARS-CoV PLpro UBL domain upstream of the catalytic site resulted in loss of antagonism (15). Further studies are required to clarify the role played by the UBL domain in PLpro-mediated IFN antagonism.

In summary, our findings revealed the multilayered targets of SARS-CoV-2 PLpro in RLR signaling pathway and implied that this critical protease encoded by SARS-CoV-2 functions in both a deubiquitination-dependent and deubiquitination-independent manner, providing valuable insights into SARS-CoV-2 replication and pathogenesis. These findings might be used for developing new strategies targeting PLpro for the effective control of SARS-CoV-2 infection.

## Data availability statement

The raw data supporting the conclusions of this article will be made available by the authors, without undue reservation.

## Author contributions

X-HR, J-WZ, Y-YC, and R-ZN performed experiments and analyzed the data. DM conceived and designed the experiments. X-HR and DM analyzed the data and wrote the manuscript. All authors read and approved the final manuscript.

## Funding

This work was supported by grants from the Scientific and Technological Research Program of Chongqing Municipal Education Commission (KJQN202000424) and Chongqing Natural Science Foundation (cstc2021jcyj-msxmX0253).

## Conflict of interest

The authors declare that the research was conducted in the absence of any commercial or financial relationships that could be construed as a potential conflict of interest.

## Publisher’s note

All claims expressed in this article are solely those of the authors and do not necessarily represent those of their affiliated organizations, or those of the publisher, the editors and the reviewers. Any product that may be evaluated in this article, or claim that may be made by its manufacturer, is not guaranteed or endorsed by the publisher.
